# Oral microbiota and dental caries data from monozygotic and dizygotic twin children

**DOI:** 10.1038/s41597-020-00691-z

**Published:** 2020-10-13

**Authors:** Yelda Kasimoglu, Mine Koruyucu, Sinem Birant, Ilker Karacan, Nursen Topcuoglu, Elif Bahar Tuna, Koray Gencay, Figen Seymen

**Affiliations:** 1 Department of Pedodontics, Faculty of Dentistry, Istanbul University, Istanbul, Turkey; 2 Department of Pedodontics, Faculty of Dentistry, Istanbul University Cerrahpasa, Istanbul, Turkey; 3 Department of Molecular Biology and Genetics, Faculty of Engineering and Natural Sciences, Istanbul Medeniyet University, Istanbul, Turkey; 4 Department of Microbiology, Faculty of Dentistry, Istanbul University, Istanbul, Turkey

**Keywords:** Paediatric dentistry, Oral microbiology

## Abstract

There are recent studies which aimed to detect the inheritance on the etiology of dental caries exploring oral composition. We present data on the oral microbiota and its relation with dental caries and other factors in monozygotic (MZ) and dizygotic (DZ) twin children. Following clinical investigation, DNA samples were collected and isolated from saliva of 198 patients (49 MZ and 50 DZ twins) with an average age of 9.7 ± 2.7 years. Salivary bacterial microbiota analysis was performed using high throughput amplicon sequencing method targeting V3-V4 region of the 16S rRNA gene. A total of 8,297,859 raw reads corresponding to 41,908 reads per sample were obtained on average. The QIIME2-deblur workflow was used for 16S rRNA amplicon analysis. Microbiome similarity analyses between twins (based on Bray-Curtis dissimilarity, weighted and unweighted Unifrac distances) showed that monozygotic twins share more bacterial microbial content compared to dizygotic twins. This is a large microbial community dataset of MZ and DZ twins with or without dental findings which can be further used for children oral microbiome profile explorations.

## Abstract (summary)

**Table:** 

Measurement(s)	Oral Microbiome • DNA • dental health
Technology Type(s)	twin design • amplicon sequencing • Examination
Factor Type(s)	zygosity status • age • sex
Sample Characteristic - Organism	Microbiota • Homo sapiens
Sample Characteristic - Environment	saliva

Machine-accessible metadata file describing the reported data: 10.6084/m9.figshare.12987797

## Background & Summary

The study of twins is used to investigate the genetic contributions to phenotypic features and diseases in human. Monozygotic twins (MZ) share the same genome, whereas dizygotic twins (DZ) on average share only half of their genome sequence. Therefore, by assuming that both types of twins have been sampled from the gene pool and that similar environmental factors set upon them, one can estimate the relative contributions of genetic and environmental influence to observe variations in different features or traits^1^.

Saliva is a body fluid that contains a variety of micro-organisms from different oral sites, such as the surfaces of tooth, tongue, buccal mucosa or gingival sulci. Although it does not contain the adherent biofilm micro-organisms of oral species, saliva is widely used as a diagnostic material, because it is easy to obtain with noninvasive techniques in sufficient quantities, store and transport^2–4^. Although there are promising studies about the potential for the salivary microbiome to become an indicator of disease diagnosis, further contributing studies are still needed^5–9^.

Caries susceptibility may be explained by the relative over abundance and/or under abundance in levels of disease-associated and health-associated microbiota^10^. Certain components of microbiota, especially *Streptococcus mutans* and *Streptococcus sobrinus*, are known as key etiological factors in the initiation and progression of caries in oral cavity^11^.

There is evidence from twin studies that genetic factors contribute to the salivary levels of *S. mutans*^12–14^. Beside this, mounting evidence exists supporting the claim that *S. mutans* is not the sole pathogenic agent responsible for carious lesions^15–21^. More than 700 bacterial species reside in human oral cavity but less than 60% of oral metagenomic reads can be mapped to reference databases at species-level^22,23^. Recent studies aim to detect the inheritance and environmental factors on the etiology of dental caries by exploring microbial composition using next generation technology^24,25^. However, exploration of microbiota and dental health association requires consideration of detailed clinical metadata together with microbial profiles.

We present data on the oral microbiota and its relation with dental caries and other factors in MZ and DZ twin children. After clinical investigation, DNA samples were collected and isolated from saliva of 198 twin children (98 monozygotic and 100 dizygotic) with an average age of 9.7 ± 2.7 years. Salivary bacterial microbiota analysis was performed using high throughput amplicon sequencing method targeting V3-V4 region of the 16S rRNA gene. A total of 8,297,859 DNA fragments belong to the V3–V4 region of the 16S rRNA gene were obtained from the 198 samples, corresponding to 41,908 ± 11,700 reads per sample on average.

**Fig. 1:**
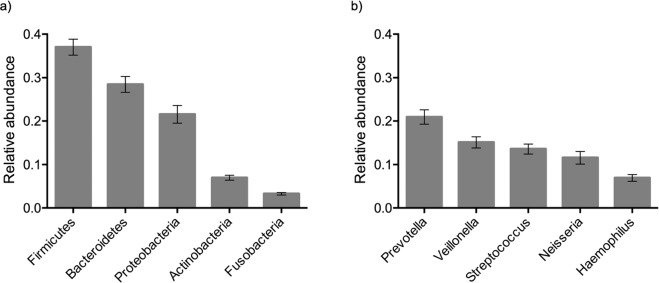
Relative phylum (**a**) and genus (**b**) level abundances among 99 twins. Error bars indicate 95% confidence intervals.

A total of 2,469 amplicon sequence variants (ASV) were detected using QIIME2 analysis. Average ASV count was 191 ± 45 per sample. Top five phylum and genus level taxonomic composition which correspond to 97.3% and 68.1% of total bacterial abundances of all samples were given in Fig. 1.

Herein, we present amplicon sequencing data for 99 twin pairs. Understanding the genetic mechanism that controls the colonization of beneficial oral microbiota could help to protect the host from dental caries and periodontal diseases. These data will be a valuable resource for oral microbiology and can be shared and re-used by the research community to investigate questions of how ecological factors modulates the oral microbiota in children towards the development of dental health and diseases. It is important to identify the healthy oral microbiota and understand the mechanism behind to develop preventive strategies, especially in children.

## Methods

### Study design

The study was approved by the Ethical Committee of the Istanbul University Faculty of Dentistry (No:2014-278) and each patient’s parents provided written informed consent. Children between 3–15 years old without a history of any systemic disease who did not use any medication that reduces saliva flow, or who were not exposed to antimicrobials in the previous three months were included voluntarily to the study.

### Clinical examination

The teeth were examined under light after dried with cotton rolls. Caries experience was measured using the Decayed, Missing, and Filled Surfaces (DMFS) index^26^. The DMFS index is applied to the permanent dentition and dmfs index expresses the number of affected tooth surfaces in primary dentition. DMFS ranges zero to 128 with molars and premolars having 5 surfaces and incisors and canines having 4 surfaces. This index accounts for teeth that are restored and missing, and those that are decayed^27^.

Periodontal health of the participants were assessed using Plaque Index^28^ (PI) given by Silness and Löe, Gingival Index^28^ (GI) given by Löe and Silness, and Bleeding on Probing^29^ (BoP) index given by Ainamo and Bay.

Oral hygiene status was assessed by the PI, the extent of plaque was measured on the buccal surfaces of the teeth and each surface was scored. According to this index, the number of 0 indicates the absence of dental plaque, and the scores between 1–3 indicate the increased presence of dental plaque. Score 0 means no plaque when the explorer is moved across the marginal gingiva. In score 1, the plaque is visible, but when the explorer is moved across the marginal gingiva very little plaques are seen. In score 2, the plaque is visible, the presence of plaque on the tooth surface as a continuous strip along the marginal gingiva. In score 3, the presence of plaque along the marginal gingiva that fills the tooth surface and extends towards the midline, filling the interproximal region^28^.

Second, the GI was used to evaluate gingival bleeding, which is the main finding of inflammation. GI scores were taken of the mesial, distal, buccal and lingual margins of the tooth. The score from each surface of the tooth was divided by total number of surfaces. According to this index, the number of 0 indicates the absence of gingival inflammation, and the scores between 1–3 indicate the increased presence of gingival inflammation, color change of the gingiva and bleeding. A score of 0 refers to healthy gingiva without signs of inflammation. Score 1 indicates mild discoloration, edema and inflammation, but no spontaneous bleeding or bleeding on probing. Score 2 indicates moderate red discoloration, edema and inflammation, without spontaneous bleeding, but bleeding on probing. In score 3, besides the presence of severe redness, edema and inflammation, spontaneous bleeding is also present^28^.

For the BoP index, gingival bleeding was recorded as present in a period of 10 seconds after with a periodontal probe. Bleeding areas were recorded as positive (=1), non-bleeding areas were recorded as negative (=0). A total of six measurements around for each tooth teeth were performed. The number of areas with bleeding was divided by the total number of areas examined and multiplied by 100 to score in percent^29^.

Gender, age, zygosity, type of delivery, and duration of breast-feeding were recorded.

### Sample collection and DNA extraction

Each individual was informed to refrain from eating, drinking or cleaning their teeth 2 hours before the examination and sampling. Saliva samples were collected using Saliva DNA Collection and Preservation Devices (Norgen Biotek, CA) according to instructions of the manufacturer. Maximal 2 ml of stimulated saliva samples were collected and preserved at room temperature until DNA extraction. DNA extraction from 500 µl preserved saliva was carried out using Saliva DNA Isolation Kit (Norgen Biotek, CA). Extracted and quantified DNA was stored at −20 °C until further analysis.

### Amplification of 16S rRNA gene, library preparation and sequencing

DNA samples were prepared for 300 bp paired end sequencing on MiSeq instrument (Illumina, USA). Each sample was first amplified using dual-indexed fusion primers targeting the hyper variable V3–V4 region of the bacterial 16S rRNA gene. PCR amplification and library preparation was performed using gene specific primers (Bakt_341F: 5′-CCTACGGGNGGCWGCAG-3′ and Bakt_805R: 5′-GACTACHVGGGTATCTAATCC-3′)^30^ attached to adaptors and multiplex identifier sequences. PCR amplification and library preparation were performed using Phusion High-Fidelity DNA Polymerase. Amplicons were cleaned and normalized using Invitrogen SequalPrep 96-well Plate Kit, then pooled in equimolar ratio and sequenced in a MiSeq instrument (Illumina, USA) with v3 chemistry, which allows 300 bp paired-end reads.

### Sequencing data processing

Demultiplexing and clipping of sequence adapters from raw sequences were performed by CASAVA data analysis software (Illumina, USA). The fragments with any mismatches to the barcodes or primers were excluded. After trimming the barcode and primer sequences from the reads, paired-end reads were merged using ‘vsearch join-pairs’ and quality filtered using ‘quality-filter q-score-joined’ within QIIME2^31^. Sequences were denoised using deblur^32^ with–p-trim-length parameter of 400 and–p-min-reads parameter of 1. Taxonomy was assigned to ASVs using ‘feature-classifier classify-sklearn’ plugin against the pre-trained Naive Bayes classifier (classifier_silva_132_99_16S_V3.V4_341F_805R.qza)^33^. Bray-Curtis dissimilarity index, weighted and unweighted distance matrixes between twins were generated using ‘diversity core-metrics-phylogenetic’ plugin with–p-sampling-depth parameter of 2,399 to ensure even sampling depth when evaluating diversity.

### Data records

The study data includes four data types, raw data, metadata (Figshare File F1), ASVs table (Figshare File F2), and ASVs sequences (Figshare File F3)^34^. Raw sequencing data is available at the National Centre for Biotechnology Information, Sequence Read Archive (SRA)^35^ while the other data files are available at Figshare^34^.

### Raw sequencing data

The paired-end 16S rRNA sequencing data were deposited in the Sequence Read Archive (SRA) with an accession number PRJNA613586^35^. The data comprises 396 FASTQ sequence files and there are two files (regarding forward and reverse reads) for each sample.

### Samples metadata, ASVs Table and ASVs sequences

Metadata of the studied samples were presented in the Figshare File F1^34^. Metadata information includes sex, age (in years), zygosity status (MZ or DZ), birth type (vaginal or C-section), breast-feeding duration (as months), as well as dental parameters such as caries status, plaque and gingival indexes and bleeding on probing. ASV table (feature table in QIIME2) including read counts per sample is also presented with taxonomy classifications in the Figshare File F2^34^. DNA sequences of ASVs were presented as fasta format in the Figshare File F3^34^.

## Technical Validation

The oral examination of thirty children were recorded by two examiners for calibration (YK, MK). The kappa value was found to be >97%, indicating a perfect agreement between the examiners. Multiple checks were done for clinical data entry and anonymous sample numbering. Zygosity status of twins were initially recorded as families’ statements. First, twins with different gender were accepted as dizygotic. Zygosity verification was performed by analysing 16 STR markers using AmpFLSTR Identifiler PCR Amplification Kit (Applied Bio, USA) and subsequent fragment analysis.

Concentration and quality assessment of the extracted genomic DNA samples were carried out using NanoDrop 2000 (Thermo Fisher Scientific, USA) spectrophotometer. OD_260/280_ and OD_260/230_ ratios were used for DNA purity evaluation. Successful amplification of the V3-V4 region of the 16S rRNA gene was verified by running and visualization of the expected bands on agarose gel. Additionally, negative (PCR reaction without template) control was performed to ensure purity of the reagents. All PCRs were performed in duplicate to reduce PCR bias. The sequencing library quality was evaluated using the Qubit Fluorometer (Thermo Fisher Scientific, USA). Raw sequencing data were first subjected to general quality control using FASTQC software^36^ before and after merging the forward and reverse reads. After merging the reads with overlapping sequences, average read length of all data was 460 ± 12 bp as expected from 341 F/805 R amplification assay.

**Fig. 2:**
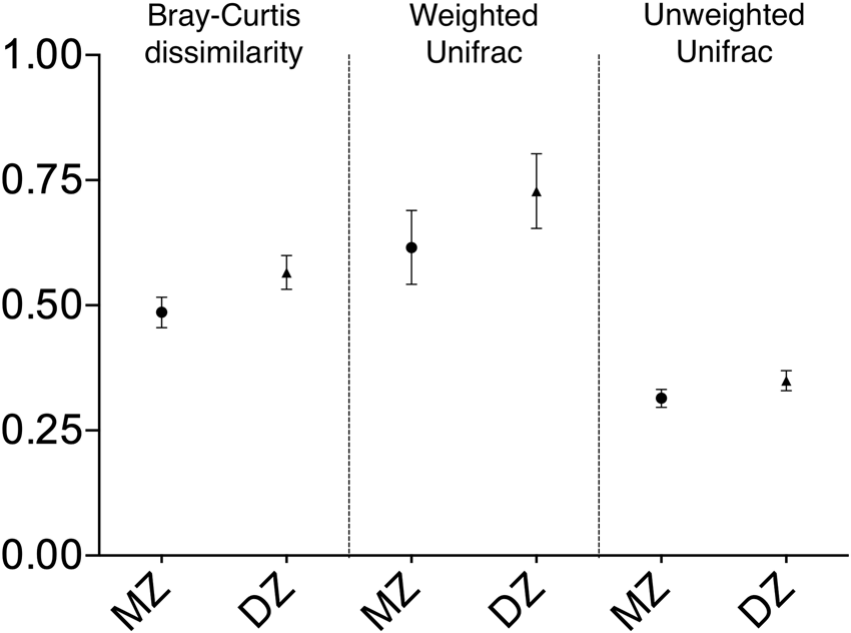
Averages of Bray-Curtis dissimilarity indices, weighted and unweighted Unifrac distances between twins (49 monozygotic and 50 dizygotic twins). Data is expressed as mean and error bars indicate 95% confidence intervals. MZ: Monozygotic twins, DZ: Dizygotic twins.

The microbiome similarity between each twin pair was determined using Bray-Curtis dissimilarity, weighted and unweighted UniFrac distances based on feature table (Figshare File F2) generated within QIIME2. Similarity analyses verified that monozygotic twins showed significantly similar bacterial microbiota profile compared to dizygotic twins (Table 1 and Fig. 2). Similar to our study, Zheng *et al*.^24^ also reported that microbial composition showed higher similarity in MZ twins than DZ twins.

**Table 1:** Comparison of mean and standard deviation of Bray-Curtis dissimilarity, weighted and unweighted distances between monozygotic and dizygotic twins.

Group	Bray-Curtis dissimilarity		Weighted Unifrac distance		Unweighted Unifrac distance	
	Mean (SD)	P value	Mean (SD)	P value	Mean (SD)	P value
Monozygotic (n = 49)	0.49 (0.11)	**0.001**	0.62 (0.26)	**0.034**	0.31 (0.06)	**0.010**
Dizygotic (n = 50)	0.57 (0.12)		0.73 (0.26)		0.35 (0.07)	

SD: Standard deviation.
